# Recurrent Gastrointestinal Symptoms and Diagnosis of Superior Mesenteric Artery Syndrome

**DOI:** 10.7759/cureus.72996

**Published:** 2024-11-04

**Authors:** Muhammad Ansar, Maham Shahbaz, Abdul Nabi Saleemi, Bruce Fox

**Affiliations:** 1 Internal Medicine, University Hospitals Plymouth NHS Trust, Plymouth, GBR; 2 Radiology, University Hospitals Plymouth NHS Trust, Plymouth, GBR

**Keywords:** anatomical variability, medical weight loss, nausea and vomiting, parenteral, superior mesenteric artery syndrome

## Abstract

This is a case of a young lady who was admitted multiple times with complaints of inability to digest food and abdominal pain. She had nausea and vomiting for long periods and was unable to tolerate orally. As she was unable to tolerate oral feeding and losing weight, she was started on nasogastric feed (NG feed) and later percutaneous endoscopic gastrostomy (PEG) tube feeding because NG feed was uncomfortable for the patient and did not alleviate the problem. Clinicians decided to look for alternative causes and an esophagogastroduodenoscopy was performed but no cause was found. Later, fluoroscopy findings favored superior mesenteric artery syndrome. Thus, the feeding tube was changed to percutaneous endoscopic gastrointestinal-jejunal (PEG-J) tube feeding which helped her with her symptoms, and the patient improved clinically after some time and gained weight as well.

This case highlights the significance of early suspicion and diagnosis of superior mesenteric artery syndrome. Timely recognition may lead to early diagnosis and management, saving patients from debilitating adverse events.

## Introduction

Superior mesenteric artery syndrome is a rare syndrome and can lead to potentially unwanted health problems if not diagnosed and treated in time. This anatomic anomaly develops due to inadequate fat pad of the Duodenum. It thus allows compression of the third part of the Duodenum between the superior mesenteric artery and abdominal aorta [1]. The majority of cases are asymptomatic unless they progress to a stage causing clinical instability. This deficiency of visceral fat deposition leads to a narrow angle of 6 to 25 degrees instead of 38 to 56 degrees between a superior mesenteric artery and the abdominal aorta [2]. It is a debilitating problem for adolescents and families as patients keep getting vomiting and abdominal pain and assessments do not clearly show causative factors. These symptoms usually lead to a vicious circle of ongoing nausea and vomiting and recurrent hospital admissions. If undiagnosed, it may lead to unnecessary scans and investigations excluding abdominal emergencies. Early suspicions, diagnosis, and management have promising results as patients adopt weight gain strategies which are helpful in the definitive management of this anatomic anomalous syndrome [3].

## Case presentation

This young patient was having recurrent admissions for abdominal symptoms such as vomiting, abdominal pain, and continuing to lose weight. Her symptoms started after eating usually within an hour. Oral and intravenous antiemetics had minimal effects. She was seen and investigated for causes of recurrent admissions starting with blood tests and ultrasound scans. However, no causative factor was found as routine investigations were normal including x-ray of the abdomen and blood tests. Furthermore, esophagogastroduodenoscopy was also performed but there were no aberrant findings. This patient continued to have vomiting and was unable to keep fluids and food.

Later, a nasogastric tube was placed for feeding but it did not resolve the issues due to still being symptomatic with nausea and abdominal pains. Further investigation with a small bowel MRI did not find any abnormality at one point and the patient remained symptomatic. Additionally, the patient’s stomach was distended at one point and the naso-jejunal tube was inserted and seemed to work for her symptoms but could not be tolerated well. Later, a percutaneous endoscopic gastrostomy (PEG) tube was placed for feeding. It seemed to solve the feeding problem but not completely.

**Figure 1:**
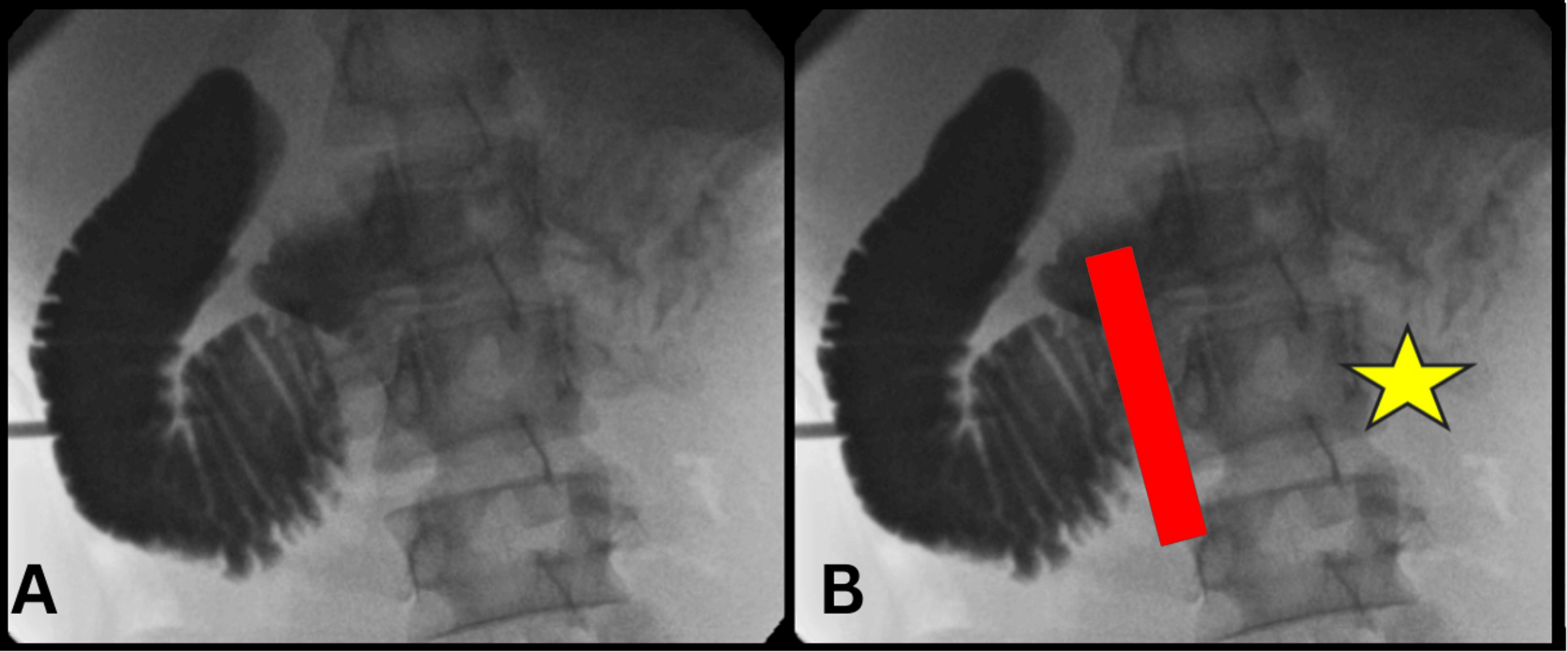
Contrast meal showing a distended second and proximal third part of the duodenum. (A) Proximal duodenum with contrast media. (B) The contrast media is not beyond the red line drawn which is a marking of the superior mesenteric artery.  The star shows the distal duodenum without contrast.

Other causes of GI disturbance were excluded. Later, a fluoroscopy stomach performed showed yo-yo reflux of the duodenum and a maximum duodenal caliber of 4.6 cm to 6 cm. This level of caliber changes and margin of the yo-yo reflux in the mid-third part of the duodenum was the main finding that led to a diagnosis of superior mesenteric artery syndrome. The fluoroscopy with contrast meal showed a distended second and proximal third part of the duodenum as shown in Figure 1A. This is where the superior mesenteric artery crosses. The contrast was not seen in the distal duodenum where contrast should have been present as shown in Figure 1B.

Then PEG tube with jejunal extension was placed and it resolved her symptoms to some extent. She was under a dietician's review and with four weeks of enteral feeding, she gained weight for the first time in months. Dieticians were in liaison with the medical team to guide about feeding. This helped her gain weight, and her symptoms improved quite significantly. Ultimately, she was discharged with a feeding regimen. She remained asymptomatic afterward. 

## Discussion

This rare anomalous disease affects about 0.13% to 3% of the general population; usually, patients are adolescents. These are labeled as cyclical vomiting syndrome and treated for this entity with antiemetics.

Many young patients get admitted to the hospital due to feeling sick and unable to keep things down. Usually, patients have to go through several investigations without any significant clues. Abdominal emergencies are generally excluded because the symptoms typically last an hour or two. This leads to further delays in diagnosis and treatment and may lead to further complications. However, rapid diagnosis and intervention benefit patients and patients recover quickly. This superior mesenteric artery syndrome is diagnosed based on imaging and clinical history only and surgical exploration is not needed to confirm the diagnosis. Few patients have been investigated with laparotomy, but it does not add much to diagnosis and management. Therefore, strong suspicion and a wide differential diagnosis help diagnose such rare anatomic pathologies [4].

**Figure 2:**
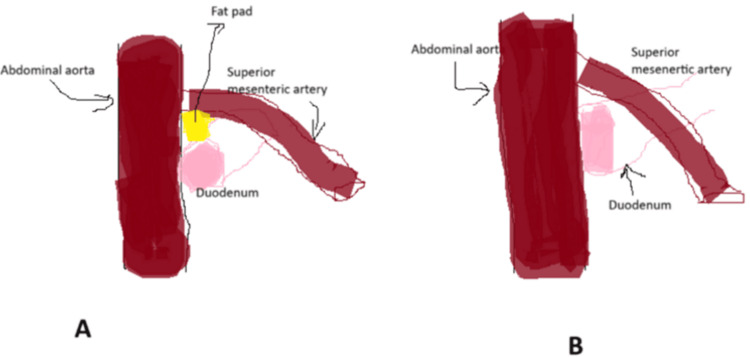
Anatomic description of superior mesenteric artery and duodenum. (A) Normal anatomy shows duodenum and superior mesenteric artery with a fat pad in between. (B) Loss of fat pad shows angulation between superior mesenteric artery and aorta and compression of duodenum.

The anatomic defect is the loss of fat pad between the abdominal aorta and the superior mesenteric artery. This leads to compression of the distal duodenum causing symptoms of gastroparesis. Figure 2A shows normal anatomical relations of the abdominal aorta and superior mesenteric artery with a fat pad in between. Figure 2B illustrates the loss of fat leading to acute angulation and compression of the duodenum.

The diagnosis is made with a team approach comprising internists, gastroenterologists, a surgical team, and pediatrics in the case of young children. The abdominal x-ray does not show much. CT scan with an angiogram might be helpful as it shows angulation and distance between the abdominal aorta and superior mesenteric artery. Fluoroscopy is also helpful in looking for contrast flow. This imaging should be correlated with clinical history, examination, and through pictures with the exclusion of other causes [5].

Usually, there is a secondary cause that may lead to weight loss, i.e., eating disorder, or celiac disease, and predisposes people to lose fat between the superior mesenteric artery and aorta. This causes a compression effect on the duodenum and leads to symptoms of abdominal pain, unable to keep things down, and leads to vomiting, abdominal distension, and pain.

## Conclusions

This case was discussed and reviewed by gastroenterology and was managed with intravenous fluids initially. However, her symptoms were not relieved. She was started on enteral feeding and later PEG feeding but to no resolution of symptoms. Additionally, fluoroscopy was performed and reported as there is compression of the duodenum and likely loss of fat pad between the superior mesenteric artery and duodenum.

Later, enteral feeding was given with a PEG tube and jejunal extension to enhance retroperitoneal fat, which may have improved the management of superior mesenteric artery syndrome. After a few weeks of this regimen, the patient got better and was discharged home.

## References

[1] Zhang ZA (2018). Superior mesenteric artery syndrome: a vicious cycle. BMJ Case Rep.

[2] Aldagher A, Almasri R, Mahmoud J (2023). Superior mesenteric artery syndrome in an 8-year-old boy: a case report. J Med Case Rep.

[3] Oliveira CA, Barbosa L, Dionísio T (2017). Superior mesenteric artery syndrome in a young woman. BMJ Case Rep.

[4] Okamoto T, Sato T, Sasaki Y (2019). Superior mesenteric artery syndrome in a healthy active adolescent. BMJ Case Rep.

[5] (2022). Superior mesenteric artery syndrome. https://rarediseases.org/rare-diseases/superior-mesenteric-artery-syndrome/.

